# Development and Characterization of Potato Starch–Pectin‐Based Active Films Enriched With Juniper Berry Essential Oil for Food Packaging Applications

**DOI:** 10.1002/fsn3.4688

**Published:** 2025-01-24

**Authors:** Saurabh Bhatia, Muhammad Jawad, Sampath Chinnam, Ahmed Al‐Harrasi, Yasir Abbas Shah, Talha Shireen Khan, Mohammed Said Al‐Azri, Esra Koca, Levent Yurdaer Aydemir, Sevgin Dıblan, Syam Mohan, Asim Najmi, Asaad Khalid, Mahbubur Rahman Khan

**Affiliations:** ^1^ Natural and Medical Sciences Research Center University of Nizwa Nizwa Oman; ^2^ School of Health Science University of Petroleum and Energy Studies Dehradun India; ^3^ Department of Chemistry M.S. Ramaiah Institute of Technology Bengaluru Karnataka India; ^4^ Department of Food Engineering Adana Alparslan Turkes Science and Technology University Adana Turkey; ^5^ Food Processing Department, Vocational School of Technical Sciences at Mersin Tarsus Organized Industrial Zone Tarsus University Tarsus Türkiye; ^6^ Substance Abuse and Toxicology Research Centre Jazan University Jazan Saudi Arabia; ^7^ Center for Global Health Research, Saveetha Medical College, and Hospitals, Saveetha Institute of Medical and Technical Sciences Saveetha University Chennai India; ^8^ Department of Pharmaceutical Chemistry and Pharmacognosy, College of Pharmacy Jazan University Jazan Saudi Arabia; ^9^ Department of Food Processing and Preservation Hajee Mohammad Danesh Science & Technology University Dinajpur Bangladesh

**Keywords:** active packaging, biopolymeric films, food packaging, natural antioxidant

## Abstract

The increasing demand for sustainable food packaging has driven the development of films based on biopolymers. However, enhancing their functional properties remains a challenge. In the current study, potato starch–pectin (PSP) composite films were fabricated and enriched with juniper berry essential oil (JBEO) to improve their physicochemical properties. The effects of incorporating different concentrations of JBEO (0.1%–1% v/v) on various properties of PSP‐based films were evaluated, including surface color, transparency, barrier properties, scanning electron microscopy (SEM), X‐ray diffraction (XRD), Fourier transform infrared spectroscopy (FTIR), thermal analysis (TGA and DTA), antioxidant activity, and antimicrobial effectiveness. Increasing the level of JBEO led to a significant decrease in the moisture content, film transparency, and mechanical attributes, while an increase in thickness, water permeability, and film elongation was observed. SEM analysis also revealed morphological properties such as some spherical, bubble‐like configuration and cracks on the surface due to an increase in JBEO concentration. TGA and DTA revealed lower weight loss in the initial cycles due to the addition of JBEO, and the thermal stability of the films improved. The antioxidant assays revealed a concentration‐dependent increase in the radical scavenging capacity of the films from 11.31% to 17.28% for DPPH and from 3.06% to 25.53% for ABTS. Moreover, significant antibacterial and antifungal activity of the bioactive films was observed against *P. aeruginosa* , *S. aureus* , and *C. albicans* . These findings suggest that JBEO enhances the functional properties of PSP films, making them suitable for active food packaging applications.

## Graphical abstract

Significant antibacterial activity of the bioactive films was observed against *P. aeruginosa* , *S. aureus* , and *C. albicans* . These findings suggest that JBEO enhances the functional and active properties of PSP edible films, making them suitable for food packaging applications.

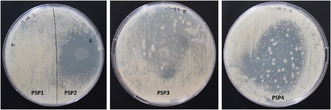

## 1 Introduction

Over the past several years, plastics have been favored for packaging due to their remarkable transparency, mechanical strength, and resistance to water and oxygen. However, the environmental concerns surrounding their nondegradable nature have grown. Additionally, there has been a noticeable shift in consumer preferences toward natural, fresh, and healthy ready‐to‐eat food items. To address the challenges of preserving perishable food products, edible films made from biopolymers have emerged as a promising solution (Dash et al. 2019). These films not only enhance the shelf life of food but also enhance its commercial appeal with a fresh, radiant appearance (Din et al. 2015; Choque‐Quispe et al. 2021).

Starch is a commonly used polymer, recognized for its superior mechanical strength and efficacy as an oxygen barrier (Muller, González‐Martínez, and Chiralt 2017). Starches derived from various sources, including potatoes, corn, sago, and rice (Torabi and MohammadiNafchi 2013; Ghasemlou et al. 2013; Al‐Hassan and Norziah 2012; Wittaya 2012), have been extensively researched for their potential functionalities, particularly within the context of food packaging. Notably, potato starch has gained attention due to its distinct attributes, such as high amylose content. Consequently, the integration of potato starch in the formulation of edible composite films might augment their overall performance characteristics (Alias and Mhd Sarbon 2019). Pectin, found abundantly in the cell walls of higher plants like citrus and apples, is among the most intricate polysaccharides in terms of structure and function (Riyamol et al. 2023). These chains mainly consist of α‐(1, 4)‐linked D‐galacturonic acid units, some of which are methyl‐esterified. While edible property of pectin has encouraged its use in preparing films, however, the resulting materials often lack robust mechanical properties (Chaichi et al. 2019; Fan et al. 2021). Therefore, the composite film can be prepared based on starch and pectin with desirable physicochemical properties.

Different essential oils have been reported for their wide range of antioxidant and antimicrobial properties (Desam et al. 2019; El‐Mesallamy et al. 2012). These oils have the potential to be incorporated in the edible films for active food packaging. Juniper berry essential oil (JBEO) is derived from the berries of the *Juniperus communis* L. plant. Traditionally, it has been employed for medicinal purposes, seasoning meat, and, most notably, as a flavoring for alcoholic drinks. JBEO's primary chemical constituents are terpenes like α‐pinene, β‐pinene, limonene, sabinene, and myrcene, which are often associated with its therapeutic qualities (Filipowicz et al. 2003; Höferl et al. 2014). Research on JBEO has highlighted its notable antibacterial and antifungal properties (Zheljazkov et al. 2018). Its usage is largely due to its significant antioxidant properties and these properties of essential oil is dependent on its concentration and type (Pandey et al. 2018; Bajac et al. 2022). JBEO has the potential to be used as a natural antioxidant and antimicrobial agent in the biopolymer‐based films for food packaging. Therefore, in the current work, films using potato starch and pectin infused with JBEO were prepared. The chemical and physical attributes, such as mechanical strength, water transmission rate, moisture content, chemical interaction between the components and color parameters, were assessed in this study. Furthermore, the antioxidant and antimicrobial features of these EO‐infused films were also examined.

## 2 Methodology

### 2.1 Materials

Potato starch and pectin were procured from Sisco Research Laboratories Private Limited, Andheri, India. Glycerol was obtained from BDH Laboratory Supplies Dorset, United Kingdom. The surfactant (Tween 80) was purchased from Merck KgaA in Gernsheim, Germany. Additionally, juniper berry essential oil (JBEO) (100 mL) with the Batch Number NNIJBEO/128/0821 was obtained from Nature Natural India in Uttar Pradesh, India.

### 2.2 Film Preparation

The potato starch/pectin composite films were developed by casting approach. Four distinct batches were prepared: PSP1 (Control), PSP2 (0.1% JBEO by volume), PSP3 (0.5% JBEO by volume), and PSP4 (1% JBEO by volume). Solutions containing 1.5% (w/v) of potato starch (PS) and pectin were separately prepared by dissolving the biopolymers in distilled water. The pectin was dissolved directly in water without heat, while the potato starch solution required heating (70°C) with stirring until it was fully dissolved. These solutions were then mixed to form the composite mixtures, designated as PSP1 to PSP4 film‐forming solutions (FFS). Each mixture received an addition of 0.5% glycerol (v/v), acting as a plasticizer, proportional to the solution volume. JBEO was incorporated into the mixtures in varying concentrations (0.1%, 0.5%, and 1%), and Tween 80 was also added as mentioned in Table 1. The prepared solutions were then cast into Petri dishes and left to dry in room conditions for 2 days. Following thorough drying, the films were carefully removed and stored in plastic bags. Films were maintained at 50% relative humidity in a test cabinet (Nüve TK 120, Türkiye) for at least 40 hours ahead of any subsequent tests.

**TABLE 1:** Composition of JBEO‐loaded potato starch/pectin (PSP) films.

Sample codes	Composition
PSP1	Potato starch (1.5%) + Pectin (1.5%) + Gly (0.5%)
PSP2	Potato starch (1.5%) + Pectin (1.5%) + Gly (0.5%) + JBEO (0.1%) + Tween 80 (0.1%)
PSP3	Potato starch (1.5%) + Pectin (1.5%) + Gly (0.5%) + JBEO (0.5%) + Tween 80 (0.5%)
PSP4	Potato starch (1.5%) + Pectin (1.5%) + Gly (0.5%) + JBEO (1%) + Tween 80 (1%)

### 2.3 Examination of Chemical Interactions

To examine the chemical interaction among the oil–biopolymer matrix, Fourier transform infrared (FTIR) spectroscopy was utilized. The FTIR spectral profiles of the films were collected using a Bruker Tensor 37 FTIR spectrometer (Ettlingen, Germany), operating in the infrared region of 4000–400 cm^−1^. The resolution of FTIR spectroscopy was set to 4 cm^−1^, which allows for sufficient detail in the spectral data to effectively analyze the chemical interaction between the oil–biopolymer matrix.

### 2.4 X‐Ray Diffraction Examination

X‐ray diffraction (XRD) examination was performed using a Bruker D8 Discover system. The device was configured to operate at a operating target voltage of 40 kV, and the angular domain for measurement was established between 5 and 55 degrees in 2‐theta (2Ɵ) angles. The collection of data proceeded at a scan rate of 0.500 seconds for each data point, with the application of copper (Kα) radiation, which has a defined wavelength of 1.5418 Å. Additionally, the crystallinity percentages of the film samples were determined using the Diffrac.Eva software package.

### 2.5 Examination of Film Microstructures

For the analysis of the films' microstructural characteristics, scanning electron microscopy (SEM) images were obtained using a JSM6510LA microscope (Jeol, Japan) at an operational voltage of 20 kV. Sample preparation involved mounting the films onto aluminum stubs utilizing a bi‐adhesive tape. This was followed by the application of a gold coating on the film surfaces by sputter coating, enhancing their visibility for the subsequent microscopic examination.

### 2.6 Thermogravimetric Assessment

The analysis of thermogravimetric properties was performed with the aid of a TA Instruments SDTQ600 thermal analyzer (New Castle, Delaware, USA). For each assessment, 10 mg of the film sample was placed in hermetic aluminum capsules before being positioned in the analysis chamber. The film was then subjected to a controlled temperature increase from 30°C to 600°C at a steady pace of 10°C per minute, within a nitrogen‐dominant atmosphere to maintain an inert and stable testing environment. For calibration accuracy, zinc served as the standard reference metal.

### 2.7 Film Thickness and Texture Characteristics

A precision digital micrometer (Model 2046F, Mitutoyo, Japan), having a resolution of 1 μm, was used for assessing the thickness of the edible films, which is reported in millimeters (mm). Thickness evaluations were conducted at 5 varied points across each film sample, and the average thickness was calculated in millimeters. The recorded thickness values were subsequently utilized for additional analyses. The assessment of mechanical characteristics, including tensile strength and the percentage of elongation for the edible films, was conducted in alignment with the ASTM standard method D882‐95 (ASTM International 2002). This analysis utilized a texture analyzer (TA. XT plus, Stable Micro Systems, Godalming, England) equipped with a 5 kg load cell. The parameters of TS and EAB were determined from the generated stress–strain curves through the application of Exponent Connect software.

### 2.8 Water Permeability and Water Solubility

The determination of the water vapor permeability (WVP) of the fabricated films was conducted using a gravimetric approach following the methodology described by Bhatia et al. (2024). Glass containers measuring 5 cm internal diameter and a 3 cm depth were utilized for this purpose. Prior to the test procedures, the films underwent a conditioning phase at room temperature (25°C) and relative humidity (50%) for 10 days. RH of the measurement systems were adjusted using water (RH = 100%) and silica gel (RH = 0%). Films were sealed firmly over the cup containing silica gel, and the cups were weighted every hours periodically to evaluate weight gain within a day. This setup allowed the measurement of water vapor permeation through the film, as the change in weight of the container over time was recorded.

On the other hand, the evaluation of water solubility for the films was carried out using a modified version of the methodology outlined by Kim and Song (2018). The calculation of the films' water solubility percentage was performed using the equation as follows:

(1) $$\mathrm{WS}=\frac{W_{1}-W_{2}}{W_{1}}\times 100$$

In this equation:*W*_1_ is the film's initial dry weight before water exposure.*W*_2_ is its weight after immersion in water, with soluble parts removed and the film re‐dried.

### 2.9 Moisture Content and Swelling Index

The water content of film samples was analyzed through a gravimetric technique. Samples were precisely trimmed to dimensions of 30 × 40 mm, and their initial weight was documented (denoted as *W*_1_). These samples were then dehydrated at a temperature of 105°C to a point where their weight remained unchanged. The final weight of the dried samples was noted as *W*_2_, and the reduction in weight was calculated using the subsequent formula:

(2) $$\mathrm{MC}=\frac{W_{1}-W_{2}}{W_{1}}\times 100$$

*W*_1_ is the initial weight of the sample (including moisture),*W*_2_ is the weight of the sample after drying, with moisture removed.

The measurement of the swelling behavior for films loaded with oil was conducted in accordance with the procedure established by Erdem, Dıblan, and Kaya (2019). Rectangular sections of the film, each with dimensions of 2 by 2 cm, were precisely cut, and their initial mass was recorded (*W*_1_). These samples were then fully immersed in deionized water at a controlled temperature of 25°C for a period of 2 min. Following immersion, the film samples were gently blotted with a filter paper to remove the surface moisture before being reweighed (*W*_2_). The swelling ratio for the samples was determined by using formula as stated below:

(3) $$\text{Swelling Index}=\frac{W_{2}-W_{1}}{W_{1}}\times 100$$

*W*_1_ is the initial dry weight of the sample,*W*_2_ is the weight after the sample has been submerged in water.

### 2.10 Transparency and Color Attributes of the Films

The transparency of the fabricated films was evaluated using a V‐10 Plus‐ONDA‐Vis spectrophotometer (ONDA, Padova, Italy) at an optical wavelength of 550 nm, as reported by Zhao, Wang, and Liu (2022). For assessing the effect of JBEO on the color attributes of the films, a spectrophotometer CM‐5 (Konica Minolta, Tokyo, Japan) was used. The color parameters were quantified in terms of lightness (L*), red/green value (a*), and yellow/blue value (b*).

### 2.11 Antioxidant Activity

For the ABTS cation radical scavenging assay, based on Re and Pellegrini (1999), 100 mg of the film sample was mixed with 1.9 mL of a 7 mmol/L ABTS radical solution. After a 6‐minute reaction period, absorbance at 734 nm was measured to determine the percentage reduction of the ABTS radical. Similarly, for the DPPH assay, the methodology reported by Brand‐Williams, Cuvelier, and Berset (1995) was followed. In this method, 50 mg of the film samples was treated with 1.95 mL of a DPPH methanol solution. Absorbance was measured at 517 nm to quantify the antioxidant activity, expressed as the percentage of DPPH radical inhibition.

### 2.12 Evaluation of Antimicrobial Properties of Oil‐Infused Films

The antibacterial efficacy of the oil‐embedded films was evaluated using agar diffusion assay, following the methodologies proposed by Seol et al. (2009) and Matuschek, Brown, and Kahlmeter (2014). The microbial strains selected for investigation were *Staphylococcus aureus* (ATCC 25923, Gram‐positive bacteria), *Pseudomonas aeruginosa* (ATCC 27853, Gram‐negative bacteria), and *Candida albicans* (ATCC 10231, fungal strain). Activation of these bacterial cultures was accomplished by incubating them on Tryptic Soy Agar (TSA) at 35°C for an 18‐h period. For the preparation of the bacterial inoculum, a sterile saline solution (0.85% w/v NaCl) was utilized to attain a turbidity equivalent to 0.5 on the McFarland scale. The bacterial suspensions were then applied to the surface of Mueller–Hinton agar plates with the help of cotton swabs to achieve a final density of roughly 10^8^ CFU/mL.

Film samples, (10 mm diameter circles), were placed on the agar plates, with certain plates serving as negative controls that contained no antibacterial substances. This is followed by incubation of the plates at 35°C from 24 up to 48 hours. The presence of inhibition zones was noted as clear regions that formed around the film samples. These zones were measured in millimeters and the average of these measurements was calculated to determine the antibacterial potency of the films in preventing bacterial proliferation.

### 2.13 Statistical Analysis

Findings in this research were depicted as the average value plus the corresponding standard deviation (SD). All experiments were repeated at least three times, and the experimental data were expressed as mean ± standard deviation (Mean ± SD). Statistical assessments were performed utilizing the SPSS software, version 17.0, formulated by SPSS Inc., Chicago, IL, USA. One‐way analysis of variance (ANOVA) was employed to measure the statistical significance of difference among the average values. Subsequent examinations were performed applying Duncan's multiple range test, setting the threshold for statistical significance at 5%.

## 3 Results and Discussion

### 3.1 FTIR Analysis

**FIGURE 1:**
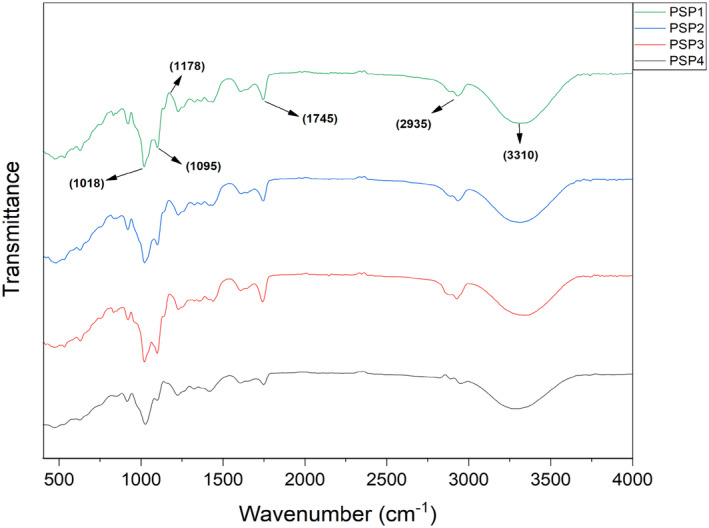
FTIR analysis of potato starch and pectin (PSP) composite edible films loaded with juniper berry essential oil (JBEO). PSP1 (control film); PSP2 (JBEO 0.1% v/v); PSP3 (JBEO 0.5% v/v); PSP4 (JBEO 1% v/v).

To investigate the chemical interactions between juniper berry oil (JBO) and film‐forming biopolymers, i.e., potato starch and pectin, Fourier transform infrared (FTIR) spectroscopy was employed. The resulting spectra, featuring characteristic peaks, are depicted in Figure 1. The acquired spectroscopic data indicates that the integration of JBO did not formed distinctive absorption bands that could signify alterations in the structural framework of the film matrix. Distinctive spectral peaks for potato starch and pectin were identified at wavenumbers 1018, 1095, 1178, 1745, 2935, and 3310 cm^−1^. The absorption bands at 1018 and 1095 cm^−1^ are indicative of C–O bond stretching vibrations. At 1178 cm^−1^, the spectrum exhibits a band corresponding to the medium stretching vibrations of the C–N bond. The pronounced peaks at 1745 and 2935 cm^−1^ are characteristic of strong C=O and medium‐intensity C–H bond stretching vibrations, respectively. Additionally, a broad absorption band at 3310 cm^−1^ was determined, which is attributed to the stretching vibrations of the –OH groups. In a previous study, FTIR analysis observed peaks around 1037 cm^−1^, which represented the C–O–C stretching vibrations of the saccharide structures in pectin and glycerol (Asfaw, Tafa, and Satheesh 2023). Previous studies have also demonstrated comparable FTIR spectroscopy profiles in edible films fabricated from potato starch and pectin (Dash et al. 2019; Choi et al. 2022).

### 3.2 X‐Ray Diffraction Analysis

**FIGURE 2:**
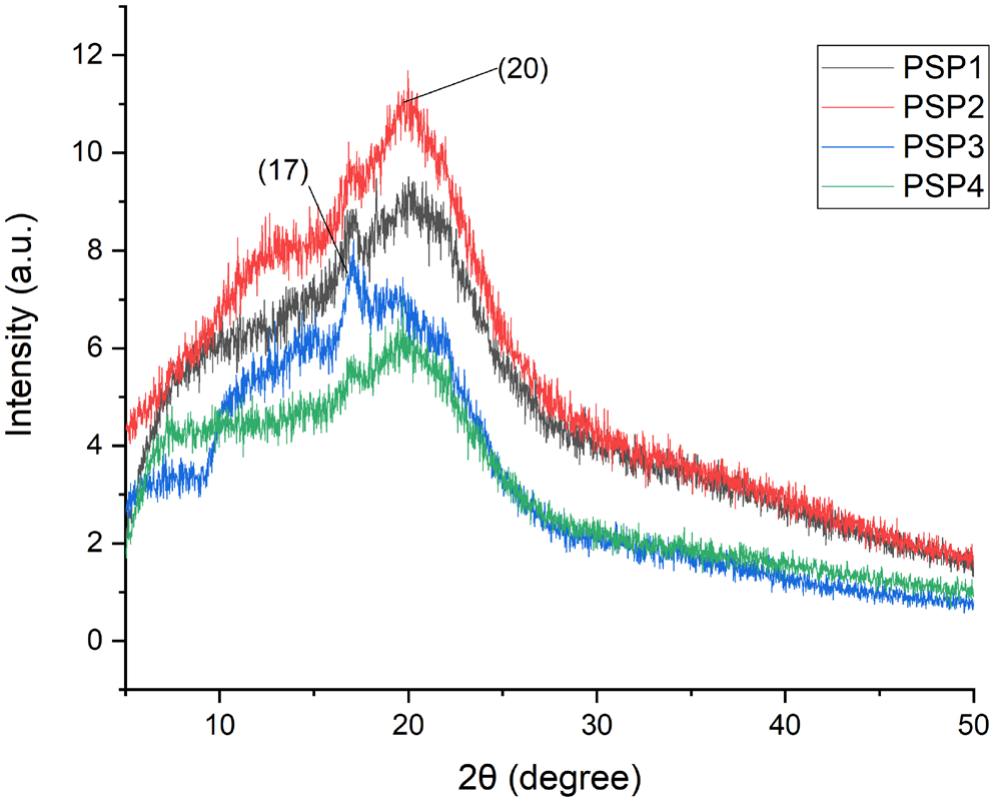
XRD diffractogram of potato starch and pectin (PSP) composite edible films loaded with juniper berry essential oil (JBEO). PSP1 (control film); PSP2 (JBEO 0.1% v/v); PSP3 (JBEO 0.5% v/v); PSP4 (JBEO 1% v/v).

The X‐ray diffraction patterns of the potato starch–pectin (PSP) edible film loaded with JBEO were obtained to confirm the crystalline structure of the composite films. Figure 2 indicates the semicrystalline nature of the PSP films. The XRD analysis revealed two peaks in the diffractogram, i.e., 2θ = 17° and 20°. The composite PSP films show an increase in the crystalline fraction as the oil is incorporated. The percentage crystallinity increases from 17% in the control PSP1 film to 34% in PSP4 (maximum concentration of EO). The characteristic peaks at 17° and 20° could be attributed to the typical B‐type crystals of starch. Almost identical patterns of X‐ray diffraction were observed in all the JBEO‐loaded PSP films as compared to control PSP films since the majority matrix of the blend films is starch and pectin. Similar results were reported by Wu et al. (2019). and Lopez‐Sanchez et al. (2017) for potato starch and pectin‐based films in their respective studies.

### 3.3 Scanning Electron Microscopy (SEM)

**FIGURE 3:**
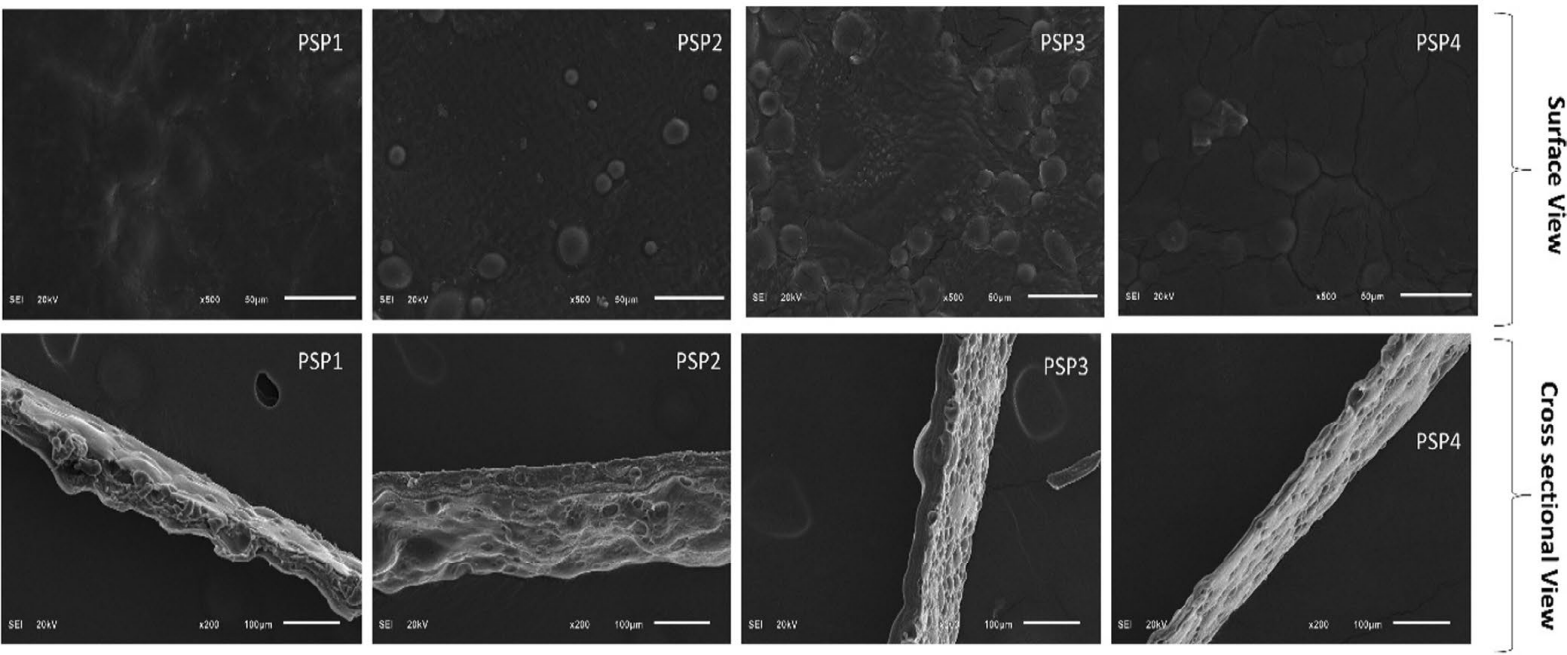
SEM analysis of potato starch and pectin (PSP) composite edible films loaded with juniper berry essential oil (JBEO). PSP1 (control film); PSP2 (JBEO 0.1% v/v); PSP3 (JBEO 0.5% v/v); PSP4 (JBEO 1% v/v).

Scanning electron microscopy (SEM) was utilized to examine the surface characteristics and the dispersion of droplets from juniper berry essential oil (JBEO) in the polymeric matrix, with the findings illustrated in Figure 3. The control films (PSP1) presented a uniform and dense surface when compared to the JBEO‐enhanced films (PSP2–PSP4). The integration of JBEO into the PSP matrix resulted in an increased occurrence of cracks on the film's surface. These cracks facilitate the movement of water from the film, resulting in increased permeability as presented in Table 2. Furthermore, the addition of JBEO was associated with the emergence of a spherical, bubble‐like configuration, which is likely due to the essential oil droplets embedded within the matrix. These observations are in line with the work of Almasi, Azizi, and Amjadi (2020), who explored the influence of nano‐emulsified marjoram (*Origanum majorana L*.) essential oil on pectin films. Additionally, similar structures have been documented for sodium alginate films embedded with essential oil nanoemulsions (NE), where the porous architecture could be ascribed to the ascent and subsequent volatilization of oil droplets during the dehydration process as observed by Acevedo‐Fani et al. (2015). It was also noted that the water vapor permeability (WVP) of the films increased with higher JBEO concentrations, a phenomenon that could be correlated with the altered morphological traits of the films, leading to enhanced moisture transmission.

**TABLE 2:** Thickness, water vapor permeability (WVP), and moisture content (MC) of PSP‐based edible films loaded with JBEO.

Sample code	Thickness (mm)	WVP ((g*mm)/(m^2^*h*kPa))	Moisture content (%)
PSP1	0.025 ± 0.006^a^	0.175 ± 0.004^a^	26.93 ± 0.07^a^
PSP2	0.033 ± 0.005^ab^	0.226 ± 0.005^b^	25.85 ± 0.06^b^
PSP3	0.036 ± 0.005^bc^	0.258 ± 0.010^c^	24.82 ± 0.39^c^
PSP4	0.043 ± 0.005^c^	0.319 ± 0.005^d^	24.27 ± 0.12^c^

*Note:* Average values placed in columns marked with different letters (^a–d^) are statistically different (*p* ≤ 0.05).

### 3.4 Thermal Analysis of PSP Samples

**FIGURE 4:**
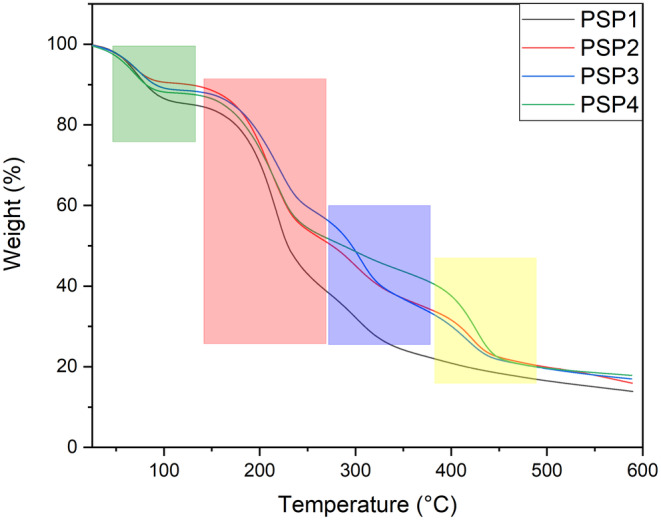
TGA thermogram of potato starch and pectin (PSP) composite edible films loaded with juniper berry essential oil (JBEO). PSP1 (control film); PSP2 (JBEO 0.1% v/v); PSP3 (JBEO 0.5% v/v); PSP4 (JBEO 1% v/v).

**FIGURE 5:**
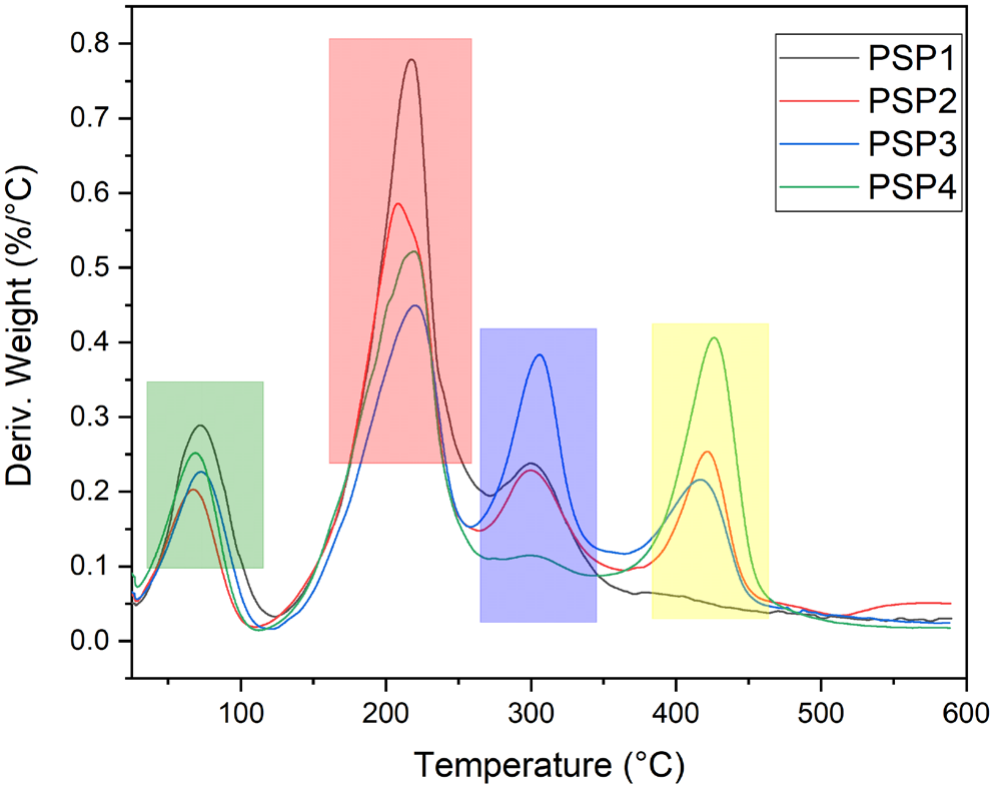
DTA thermogram of potato starch and pectin (PSP) composite edible films loaded with juniper berry essential oil (JBEO). PSP1 (control film); PSP2 (JBEO 0.1% v/v); PSP3 (JBEO 0.5% v/v); PSP4 (JBEO 1% v/v).

Thermal analysis using TGA and DTA has been conducted to assess the thermal behavior and degradation characteristics of the film materials. The findings are depicted in Figures 4 and 5, corresponding to TGA and DTA analyses. All the film samples demonstrated a multiphase weight reduction, which is evident in the TGA and DTA graphs. Initially, a decrease in weight occurring between 25°C and 120°C is attributed primarily to the loss of moisture, both surface‐bound and incorporated within the material, as reported by de Morais Lima et al. (2017); Kchaou et al. (2020). Subsequently, a prominent decline in mass between 180°C and 430°C is observed, which is largely ascribed to the thermal breakdown of both pectin and starch components. The initial phase of this segment, ranging from 200°C to 280°C, is related to pectin's decomposition, followed by a significant mass loss from 290°C to 425°C, corresponding to the structural breakdown of starch, as indicated by a previous study as well (Basiak, Lenart, and Debeaufort 2017). Films incorporated with JBEO showcased an enhanced resistance to thermal degradation in comparison to the control PSP film, which was particularly noticeable in the early stages, as shown by the DTA curve. This enhancement in thermal stability with the addition of essential oils aligns with previous research where cassava starch‐based films were studied (Zhou et al. 2021). The thermal stability could be due to the interactions within the multiphase system and the improved compatibility between the film components (Xu et al. 2019).

### 3.5 Color Analysis and Transparency

In food packaging, color plays a pivotal role as it not only satisfies consumer preferences but also determines the commercial importance of a specific food item. The Hunter scale showcases parameters like L*, a*, b*, and Δ*Ε*, which are essential for understanding color gradations. Table 3 details these color characteristics, as well as the whiteness index and overall color variance (ΔE), for films made of potato starch and pectin infused with juniper berry essential oil (JBEO). The results showed a notable rise in the yellowness (b*) when the essential oil concentration was increased. This increase in b* value could be due to the inherent yellow color of JBEO. As the concentration increases, the films tend to exhibit a mild yellow tint.

**TABLE 3:** Color parameters comprising L*, a*, b*, color difference (Δ*E*), and transmittance (%) of PSP‐based films loaded with juniper berry essential oil (JBEO).

Sample code	L*	a*	b*	Δ*E*	Transmittance (%)
PSP1	97.84 ± 0.13^a^	0.26 ± 0.01^a^	0.68 ± 0.03^a^	2.06 ± 0.13^a^	45.86 ± 1.42^a^
PSP2	98.33 ± 0.15^b^	0.29 ± 0.03^a^	0.72 ± 0.03^a^	2.54 ± 0.16^b^	34.36 ± 0.70^b^
PSP3	98.10 ± 0.25^a^ ^b^	0.57 ± 0.02^b^	1.27 ± 0.04^b^	2.59 ± 0.20^b^	28.10 ± 1.09^c^
PSP4	98.95 ± 0.79^b^	0.60 ± 0.05^b^	1.13 ± 0.12^b^	3.66 ± 0.49^c^	20.95 ± 0.86^d^

*Note:* Average values placed in columns marked with different letters (a–d) are statistically different (*p* ≤ 0.05).

The results demonstrated that the color alterations in the resultant EFs could be attributed to the color pigments present in JBEO. While films based on polysaccharides typically lack color, adding essential oils often leads to a shift in hue, specifically toward yellowness. This change might arise from the phenolic substances in the EOs, which tend to absorb light at shorter wavelengths. A study conducted by Frank et al. reported similar results as Cinnamon essential oil nanoemulsion was incorporated into alginate‐based films (Frank et al. 2018). Another related study by Iraj et al. reported similar outcomes where Melissa essential oil was used in films based on sodium caseinate (Sani, Marand, et al. 2021). Furthermore, there was a modest rise in lightness (L*) and Δ*E* in the films containing JBEO. These findings highlight the variations in the color attributes of bioactive films.

Light transmittance is a crucial optical feature of films, influencing their appearance, market appeal, and adaptability for diverse uses, including food products. Films without additives (PSP1) demonstrated higher light transmittance than those enriched with JBEO (PSP2‐PSP4), as detailed in Table 3. As the concentration of JBEO was increased, light transmittance correspondingly dropped. This could be due to the scattering effect caused by the dispersed JBEO droplets within the EFs. A similar pattern was noted in a separate study where the incorporation of lemongrass essential oil led to reduced light transmittance (Azizah et al. 2023).

### 3.6 Water Vapor Permeability (WVP)

Water vapor permeability (WVP) is a pivotal characteristic of edible films, given its role in managing moisture in packaged food. Films with low water vapor permeability can prevent excess moisture loss and provide good barrier properties, thereby helping to maintain food quality and extend shelf life. In this study, as the concentration of JBEO increased, there was a rise in the WVP of edible films, with the specifics detailed in Table 2. WVP was observed to be 0.175 (g*mm)/(m^2^*h*kPa) (PSP1), which increased to 0.319 (g*mm)/(m^2^*h*kPa) in films loaded with the maximum concentration of JBEO (PSP4). As noted in a previous study by Oliveira et al. (2016), pectin can also enhance a film's resistance to water due to its emulsifying properties. However, in this study, the pectin concentration remained constant in all samples. The observed rise in WVP might be linked to the interaction between JBEO and the film matrix's hydroxyl groups. A parallel observation was made by Nisar et al. (2018) in films made of pectin that were augmented with clove bud essential oil.

### 3.7 Moisture Content

Table 2 reveals that as the concentration of JBEO in PSP edible films increased, there was a notable reduction in moisture content (MC). The MC of the control film (PSP1) was observed at 26.93%, which decreased to 24.27% for PSP4, the film with the highest JBEO concentration. The addition of JBEO appears to improve the water resistance of PSP films by modifying how water molecules interact with the polymer chains. This observation aligns with findings from other research eports, where EO integration led to a significant MC reduction (Hasheminya et al. 2019; Dashipour et al. 2015). The study observed a reduction in MC with increasing concentrations of JBEO in the films, indicating enhanced hydrophobic properties. This reduction suggests that JBEO alters the film matrix, limiting water's ability to interact with the polymer. Conversely, the increase in WVP with higher JBEO concentrations indicates that while the films resist liquid water penetration, they may allow more water vapor to pass through due to structural modifications and increased free volume in the film matrix.

### 3.8 Thickness

Table 2 shows a rise in thickness upon incorporating JBEO. PSP1 (control film) had a thickness measurement of 0.025 mm. However, with the addition of JBEO, there was a noticeable increase. For films enriched with the highest JBEO concentration (PSP4), the thickness increased to 0.043 mm. The film's thickness plays a crucial role in measuring its water transmission rate. This increased thickness might be linked to the essential oil's water‐repellent properties and the development of microdroplets within the film's matrix (Valizadeh et al. 2019).

### 3.9 Mechanical Attributes (Tensile Strength and Elongation at Break)

In food packaging applications, one of the primary goals is to maintain a suitable tensile strength of the packaging material. To evaluate the potential of edible films for use in food packaging, tensile strength (TS) and elongation (%) are important parameters. They provide insights into the film's ability to maintain its form and function during usage.

This research aimed to understand the effects of JBEO on the mechanical attributes of PSP films. As presented in Table 4, different film samples, ranging from the control film (PSP1) to PSP films infused with JBEO (PSP2‐PSP4), exhibited varied TS and elongation (%) parameters. Specifically, TS witnessed a decline from 14.65 MPa (in the control film) to 4.40 MPa (in a film loaded with the maximum concentration of JBEO). Meanwhile, elongation at break (%) increased from 13.51% in the control PSP1 film to 18.53% in PSP4.

**TABLE 4:** Tensile strength (TS) and elongation (%) of PSP‐based edible films loaded with JBEO.

Sample code	Tensile Strength (MPa)	Elongation at break (%)
PSP1	14.65 ± 0.19^a^	13.51 ± 0.63^a^
PSP2	9.90 ± 0.74^b^	13.75 ± 0.95^a^
PSP3	7.64 ± 0.77^c^	16.13 ± 0.03^b^
PSP4	4.40 ± 0.26^d^	18.53 ± 0.78^c^

*Note:* Average values placed in columns marked with different letters (^a–d^) are statistically different of *p* ≤ 0.05.

This observed trend might be due to the limited synergistic relationship between the hydrophobic JBEO and PSP films. The introduction of JBEO to the PSP structure seems to disrupt the cohesive forces between polymer chains. Incorporating EOs or lipids into biopolymer‐based films can potentially disrupt the bond between polymer chains, resulting in the creation of more flexible zones within the film, thus impacting the EAB (Limpisophon, Tanaka, and Osako 2010). Introducing EOs tends to weaken the molecular cohesion within a biopolymer, likely interfers with the interaction of polymer chains. This interference often results in a decline in TS and an increase in the EAB (Shen and Kamdem 2015; Jamróz, Juszczak, and Kucharek 2018).

### 3.10 DPPH and ABTS Antioxidant Analysis

**FIGURE 6:**
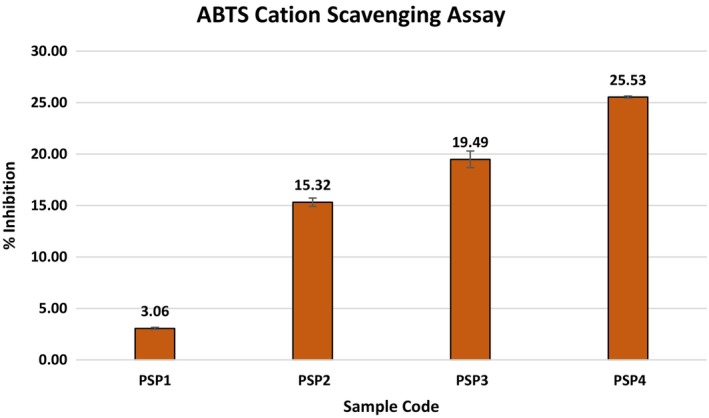
Illustration of ABTS cation scavenging activity of PSP edible films loaded with JBEO. PSP1: Control film; PSP2: JBEO (0.1% v/v); PSP3: JBEO (0.5% v/v); PSP4: JBEO (1% v/v). Error bars display standard deviation.

**FIGURE 7:**
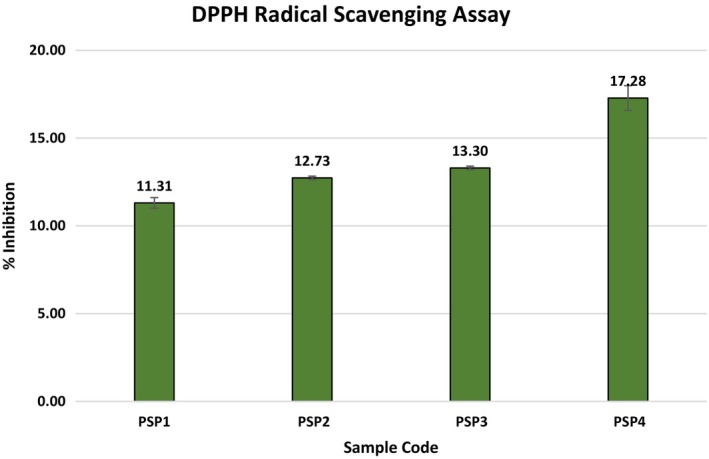
Illustration of DPPH radical scavenging activity of PSP edible films loaded with JBEO. PSP1: Control film; PSP2: JBEO (0.1% v/v); PSP3: JBEO (0.5% v/v); PSP4: JBEO (1% v/v). Error bars display standard deviation.

Films incorporated with essential oils exhibit notable antioxidant properties, serving as a natural alternative to conventional synthetic antioxidants. These films not only augment the organoleptic properties, i.e., smell, color, taste, and texture, of food but also enhance its preservation and overall quality. However, the desirability of the effects of essential oil on the sensory properties of the packed food can vary depending on the type of food and consumer preferences, making it a crucial consideration in the application of such films. In this work, the antioxidant efficacy of films loaded with JBEO within a PSP matrix was assessed through DPPH and ABTS cation radical scavenging assays. Figures 6 and 7 and illustrate a discernible rise in the radical‐neutralizing potential of the JBEO‐infused PSP films.

The film sample PSP4, fortified with the highest concentration of JBEO, displayed pronounced DPPH and ABTS cation radical neutralization capabilities, in contrast to the control film sample, which manifested minimal antioxidant properties. The integration of JBEO led to a marked amplification in the DPPH radical neutralizing capability of the film samples, registering an increase from 3.06% to 25.5%. In addition, the ABTS cation radical neutralization exhibited a significant increase from 11.31% to 17.8%. This enhanced antioxidant activity is likely due to the presence of diverse bioactive constituents and flavonoids present in JBEO. This assertion aligns with the findings of Lamer‐Zarawska (1975), Ilyas and Ilyas (1990), and Bais et al. (2014). These studies have documented that JBEO comprises an array of flavonoids such as apigenin, rutin, luteolin, quercetin‐3‐O‐arabinosyl‐glucoside, quercetin‐3‐O‐rhamnoside, quercitrin, scutellarein, nepetin, amentoflavone, and bilobetin, which are instrumental in its potent antioxidant properties (Pandey et al. 2018).

### 3.11 Antimicrobial Assay

**FIGURE 8:**
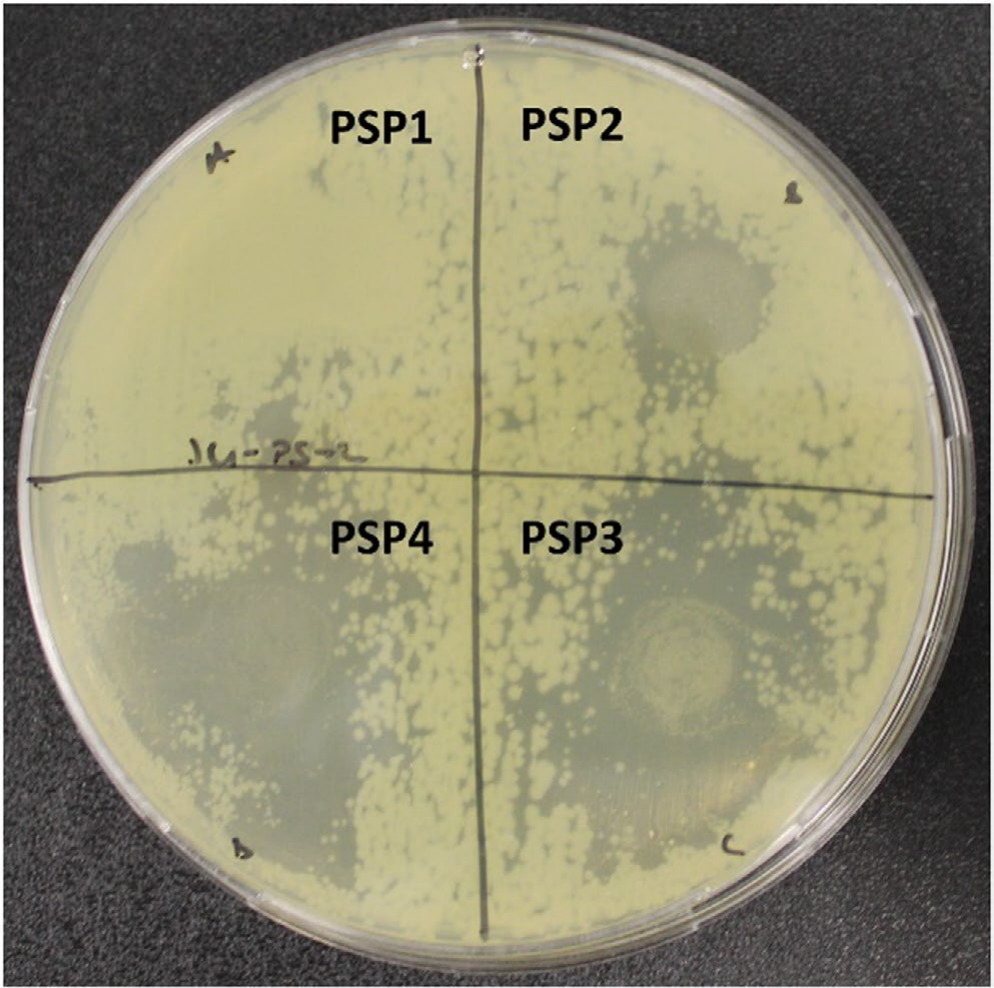
Pictograph of *Pseudomonas aeruginosa* zone of inhibition (included with disc diameter of 10 mm) PSP1: Control film, PSP2: JBEO (0.1% v/v) film, PSP3: JBEO (0.5% v/v) film, and PSP4: JBEO (1% v/v) film.

**FIGURE 9:**
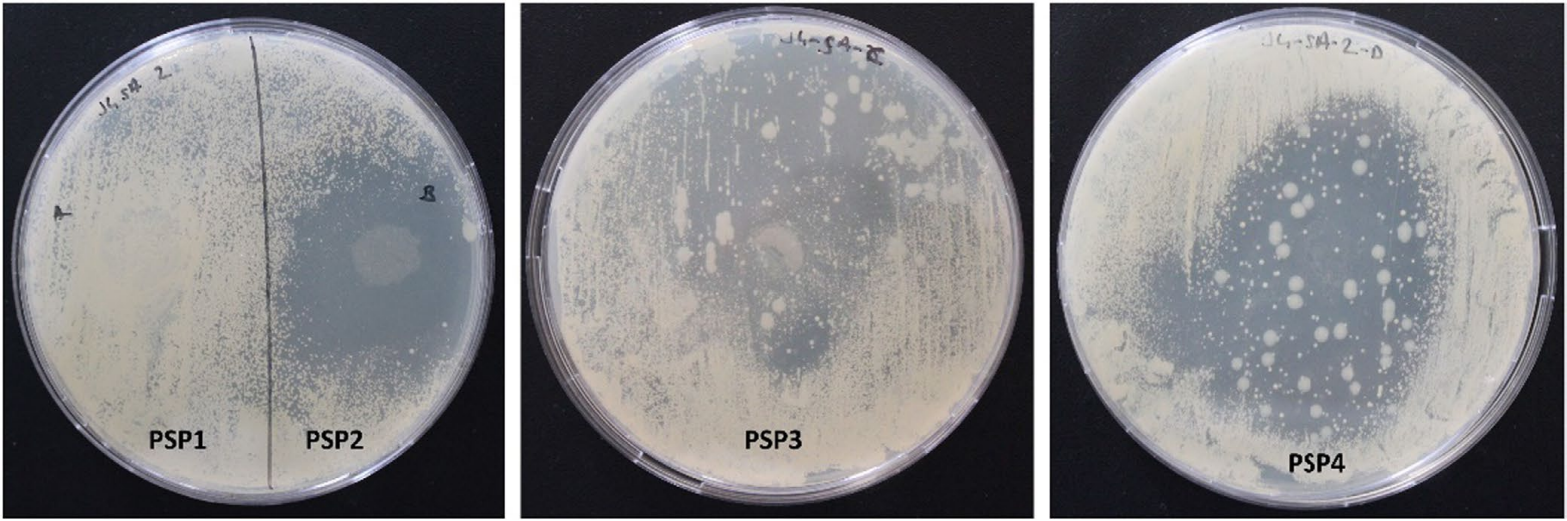
Pictograph of *Staphylococcus aureus* zone of inhibition (included with disc diameter of 10 mm) PSP1: Control film, PSP2: JBEO (0.1% v/v) film, PSP3: JBEO (0.5% v/v) film, and PSP4: JBEO (1% v/v) film.

**FIGURE 10:**
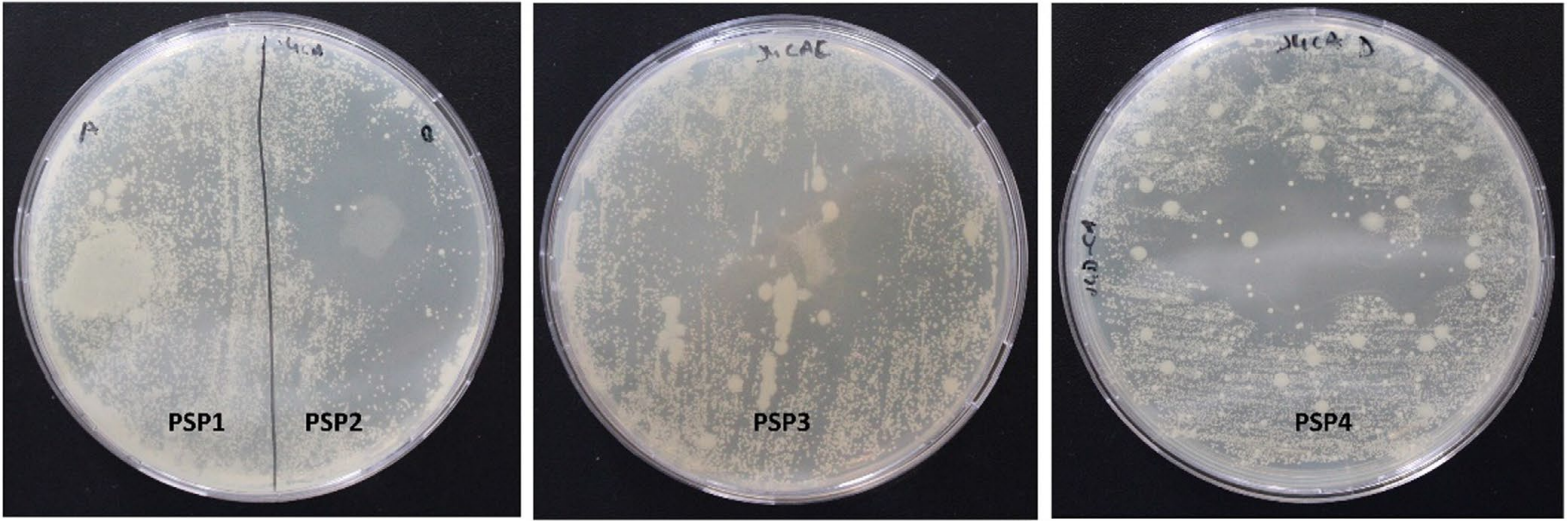
Pictograph of *Candida albicans* zone of inhibition (included with disc diameter of 10 mm). PSP1: Control film, PSP2: JBEO (0.1% v/v) film, PSP3: JBEO (0.5% v/v) film, and PSP4: JBEO (1% v/v) film.

The antimicrobial assay of JBEO‐loaded films was carried out against Gram‐negative ( *Pseudomonas aeruginosa* ) (Figure 8), Gram‐positive ( *Staphylococcus aureus* ) (Figure 9) bacterium, and fungi ( *Candida albicans* ) (Figure 10). PSP1 (control) composite films did not show any antimicrobial activity against any of the test organisms. Furthermore, the zone of inhibition (ZOI) of the JBEO‐loaded films increased in a concentration‐dependent order. Table 5 shows the antibacterial and antifungal activity of the PSP films. A maximum ZOI of 32.73, 57.15, and 43.10 mm was observed for PSP4 films against *P. aeruginosa* , *S. aureus*, and *C. albicans* , respectively. Bioactive compounds are crucial for the biological activity of juniper berry essential oil (JBEO). It contains various bioactive compounds, including monoterpene, α‐pinene, sabinene, myrcene, etc., which show antimicrobial activity. The compositions and concentrations of bioactive components may vary due to environmental factors, which can impact the biological properties of the essential oil (Ghouti et al. 2018; Esteban et al. 2023) (Najar et al. 2020). Previous studies reported similar antimicrobial results of JBEO against Gram‐positive ( *E. faecalis* and *S. aureus* ) and Gram‐negative bacteria ( *E. coli* ) (Höferl et al. 2014). Another recent study noted considerable bactericidal as well as bacteriostatic activity of JBEO against *Listeria monocytogenes* (Vasilijević et al. 2019).

**TABLE 5:** Antimicrobial activity of PSP films incorporated with juniper berry essential oil (JBEO) against test microorganisms *P. aeruginosa* (Gram‐negative), *S. aureus* (Gram‐positive), and *C. albicans* (fungi).

Sample	Zone of inhibition (mm)		
	*P. aeruginosa*	*S. aureus*	*C. albicans*
PSP1	No zone	No zone	No Zone
PSP2	14.15 ± 0.95^a^	40.96 ± 3.42^a^	40.87 ± 4.02^a^
PSP3	30.25 ± 5.98^b^	42.57 ± 1.65^a^	42.80 ± 5.85^a^
PSP4	32.73 ± 5.85^b^	57.15 ± 6.77^b^	43.10 ± 10.21^a^

Average values places in columns marked with different letters (^a–d^) are statistically different.

## 4 Conclusions

In this research, films based on potato starch/pectin (PSP) were prepared with various concentrations of JBEO. The homogeneity of JBEO distribution and its interaction with the PSP composite were key factors influencing the films' physical, microstructural, barrier, and mechanical attributes. The inclusion of JBEO was found to significantly improve the resistance of the film to thermal degradation. Mechanical testing indicated that higher levels of JBEO correlated with reduced tensile strength but increased the elongation of the films. Antioxidant assessments showed improved antioxidant activity of the films. Furthermore, the films exhibited notable antimicrobial properties, effectively inhibiting the tested pathogenic bacteria and fungi. These findings underscore the potential of these films to serve as sustainable alternatives to traditional synthetic packaging in food applications.

## Author Contributions

**Saurabh Bhatia:** conceptualization (equal), supervision (equal), writing – original draft (equal), writing – review and editing (equal). **Muhammad Jawad:** conceptualization (equal), methodology (equal), software (equal), writing – original draft (equal), writing – review and editing (equal). **Sampath Chinnam:** formal analysis (equal), validation (equal), writing – review and editing (equal). **Ahmed Al‐Harrasi:** project administration (equal), supervision (equal). **Yasir Abbas Shah:** software (equal), writing – original draft (equal), writing – review and editing (equal). **Talha Shireen Khan:** methodology (equal), software (equal), writing – review and editing (equal). **Mohammed Said Al‐Azri:** formal analysis (equal), methodology (equal), software (equal). **Esra Koca:** data curation (equal), methodology (equal), software (equal). **Levent Yurdaer Aydemir:** formal analysis (equal), methodology (equal), software (equal), writing – review and editing (equal). **Sevgin Dıblan:** methodology (equal), software (equal), writing – review and editing (equal). **Syam Mohan:** formal analysis (equal), validation (equal), writing – review and editing (equal). **Asim Najmi:** formal analysis (equal), validation (equal), writing – review and editing (equal). **Asaad Khalid:** formal analysis (equal), validation (equal), writing – review and editing (equal). **Mahbubur Rahman Khan:** data curation (equal), formal analysis (equal), writing – review and editing (equal).

## Conflicts of Interest

The authors declare no conflicts of interest.

## Data Availability

All the data will be provided on request.
